# Inhibition of Calcineurin/NFAT Signaling Blocks Oncogenic H-Ras Induced Autophagy in Primary Human Keratinocytes

**DOI:** 10.3389/fcell.2021.720111

**Published:** 2021-07-19

**Authors:** Shuangshuang Wang, Hua Qian, Liwei Zhang, Panpan Liu, Dexuan Zhuang, Qun Zhang, Fuxiang Bai, Zhihong Wang, Yonggan Yan, Jing Guo, Jun Huang, Xunwei Wu

**Affiliations:** ^1^Department of Tissue Engineering and Regeneration, School and Hospital of Stomatology, Cheeloo College of Medicine, Shandong University & Shandong Key Laboratory of Oral Tissue Regeneration and Shandong Engineering Laboratory for Dental Materials and Oral Tissue Regeneration, Jinan, China; ^2^Department of Stomatology, The Second Hospital of Shandong University, Jinan, China; ^3^Department of Pediatric Dentistry, School and Hospital of Stomatology, Cheeloo College of Medicine, Shandong University & Shandong Key Laboratory of Oral Tissue Regeneration and Shandong Engineering Laboratory for Dental Materials and Oral Tissue Regeneration, Jinan, China; ^4^Qilu Children’s Hospital of Shandong University, Jinan, China; ^5^Center for Advanced Jet Engineering Technologies, Key Laboratory of High Efficiency and Clean Mechanical Manufacture of Ministry of Education, School of Mechanical Engineering, Shandong University, Jinan, China; ^6^Department of Orthodontics, School and Hospital of Stomatology, Cheeloo College of Medicine, Shandong University & Shandong Key Laboratory of Oral Tissue Regeneration and Shandong Engineering Laboratory for Dental Materials and Oral Tissue Regeneration, Jinan, China; ^7^Savaid Stomatology School of Hangzhou Medical College, Ningbo Stomatology Hospital, Ningbo, China; ^8^Cutaneous Biology Research Center, Massachusetts General Hospital, Harvard Medical School, Boston, MA, United States

**Keywords:** H-Ras, calcineurin, NFATc1, autophagy, keratinocyte

## Abstract

Mutations of H-Ras, a member of the RAS family, are preferentially found in cutaneous squamous cell carcinomas (SCCs). H-Ras has been reported to induce autophagy, which plays an essential role in tissue homeostasis in multiple types of cancer cells and in fibroblasts, however, the potential role of H-Ras in regulating autophagy in human keratinocytes has not been reported. In this study, we found that the stable expression of the G12V mutant of H-RAS (H-Ras^*G12V*^) induced autophagy in human keratinocytes, and interestingly, the induction of autophagy was strongly blocked by inhibiting the calcineurin/nuclear factor of activated T cells (NFAT) pathway with either a calcineurin inhibitor (Cyclosporin A) or a NFAT inhibitor (VIVIT), or by the small interfering RNA (siRNA) mediated knockdown of calcineurin B1 or NFATc1 *in vitro*, as well as *in vivo.* To characterize the role of the calcineurin/NFAT pathway in H-Ras induced autophagy, we found that H-Ras^*G12V*^ promoted the nuclear translocation of NFATc1, an indication of the activation of the calcineurin/NFAT pathway, in human keratinocytes. However, activation of NFATc1 either by the forced expression of NFATc1 or by treatment with phenformin, an AMPK activator, did not increase the formation of autophagy in human keratinocytes. Further study revealed that inhibiting the calcineurin/NFAT pathway actually suppressed H-Ras expression in H-Ras^*G12V*^ overexpressing cells. Finally, chromatin immunoprecipitation (ChIP) assays showed that NFATc1 potentially binds the promoter region of H-Ras and the binding efficiency was significantly enhanced by the overexpression of H-Ras^*G12V*^, which was abolished by treatment with the calcineurin/NFAT pathway inhibitors cyclosporine A (CsA) or VIVIT. Taking these data together, the present study demonstrates that the calcineurin/NFAT signaling pathway controls H-Ras expression and interacts with the H-Ras pathway, involving the regulation of H-Ras induced autophagy in human keratinocytes.

## Introduction

Hyperactive retrovirus-associated DNA sequence (Ras) gene mutations are frequently found in human cancers and are associated with cancer pathogenesis. Normally, Ras is regulated to cycle between the active and inactive states, however, the oncogenic mutant Ras, usually a point mutation at residues 12 or 61, only binds GTP in an uncontrolled manner, and is, therefore, constitutively active (Downward, 2003; Nussinov et al., 2018). Around 30% of human cancers have constitutively activating mutations of Ras genes plus frequent aberrations in other part of the Ras pathways, so the abnormal function of Ras and its related signaling pathways is one of the most transformative events in human cancers (Bos, 1989). Mutations of H-Ras, a member of the RAS family, are preferentially found in cutaneous squamous cell carcinomas (SCCs). Previous studies have shown that oncogenic H-Ras can induce proliferative arrest or senescence or differentiation, depending on the cellular context (Sarkisian et al., 2007; Wu et al., 2010). H-Ras has been also shown to play a role either in the positive or negative regulation of autophagy, depending on the cell type and the signaling context (Schmukler et al., 2014). Autophagy is a highly conserved cellular mechanism that degrades unnecessary or damaged components within cells (Cao et al., 2021). The induction of autophagy usually benefits cell survival, however, it can also result in cell death, called autophagic cell death, or non-apoptotic cell death (type II programmed cell death; Codogno and Meijer, 2005). More and more evidence has shown that autophagy is involved in various biological functions, including cancer formation and progression, and autophagy has been shown to play both anti- and pro-tumorigenic roles, depending on the tumor type, stage and cellular context (Schmukler et al., 2014). H-Ras^*G12V*^ was reported to inhibit autophagy in IEC-18 intestinal epithelial cells through degradation of the autophagic gene beclin-1 (Yoo et al., 2010). However, the overexpression of H-Ras^*G12V*^ was mainly reported to induce autophagy in non-tumorigenic or cancer cell lines. For instance, the overexpression of H-Ras^*G12V*^ can induce autophagy in NIH3T3 and Rat2 fibroblasts (Byun et al., 2009; Wu et al., 2011). Mutated H-Ras can also enhance autophagy to trigger cell death in several human cancer cell lines, such as the glioma cell line U251 and the gastric cancer line MKN-1 (Chi et al., 1999). Although H-Ras is frequently mutated in skin SCCs (Su et al., 2012), little is known about the role of H-Ras in autophagy in keratinocytes.

Calcineurin is the only known serine/threonine phosphatase under calcium/calmodulin control (Mammucari et al., 2005). Calcineurin is a heterodimeric enzyme formed by a catalytic subunit (Calcineurin A, CnA) and a Ca^2+^-binding regulatory subunit (Calcineurin B, CnB). CnB is expressed in two isoforms, CnB1 (ubiquitous) and CnB2 (testis-specific) (Mammucari et al., 2005). Among proteins that are dephosphorylated as a consequence of Calcineurin activation are nuclear factor of activated T cells (NFAT), which contains 4 isoforms, NFATc1, NFATc2, NFATc3, and NFATc4 (Crabtree and Olson, 2002). The activity of each specific NFAT isoform is directly regulated by the phosphatase calcineurin (CN), which dephosphorylates NFAT to induce its nuclear translocation and transcriptional activity. Calcineurin/NFAT signaling plays an important role in controlling cells of the immune system, specifically T cells (Crabtree and Olson, 2002). Cyclosporine A (CsA), which suppresses calcineurin activity (Schreiber, 1991; Bendickova et al., 2017), is widely used for immune suppression in a number of clinical conditions, including the therapy of a variety of dermatological diseases and as an immunosuppressant for transplant patients (Allison, 2000). An important side effect of CsA treatment is the formation of aggressive skin SCCs. SCCs are the most common skin cancer in transplant recipients, occurring 65–250 times as frequently as in the general population (Hartevelt et al., 1990; Jensen et al., 2000; Lindelof et al., 2000; Kempf et al., 2012). Several mechanisms responsible for the highly aggressive behavior of SCCs that develop in these patients have been suggested, such as increases in the secretion of transforming growth factor-b and vascular endothelial growth factor conducive to SCC formation (Hofbauer, 2010). We previously found that calcineurin/NFAT inhibitors can promote the tumorigenesis of keratinocytes expressing H-Ras^*G12V*^ by inhibiting cellular senescence and differentiation, which is induced by the oncogene H-Ras as a safeguard mechanism (Wu et al., 2010). Therefore, it would be interesting to know whether the calcineurin/NFAT pathway involved in autophagy is potentially regulated by oncogenic mutant H-Ras in keratinocytes.

In the present study, we found that overexpression of the oncogene H-Ras^*G12V*^ can induce autophagy in human keratinocytes, and that calcineurin/NAFT inhibitors can block the H-Ras^*G12V*^ induced autophagy. Our findings demonstrate that the oncogene H-Ras^*G12V*^ can coordinate with the calcineurin/NFAT pathway to regulate autophagy in human keratinocytes.

## Materials and Methods

### Human Keratinocyte Culture and Reagents

Primary human keratinocytes were isolated from foreskin tissues and were cultured in serum-free keratinocyte medium (k-SFM) (Thermo Fisher Scientific, Waltham, MA, United States, #17005042) as previously described (Wu et al., 2014; Wen et al., 2018; Qian et al., 2019). The procedure for obtaining human foreskin tissues from discarded hospital specimens without any personal identity information was approved by the Medical Ethical Committee of the School of Stomatology Shandong University (No. GR201711, Date: 02-27-2017). Early passage (passage 2 or 3) keratinocytes were used for all experiments. CsA (Sigma-Aldrich, Darmstadt, Germany, #30024) was dissolved in DMSO and stored as a stock solution (10 mM). NFAT inhibitor VIVIT (*RRRRRRRRRRRGGGMAGPHPVIVITGPHEE*) peptides and control VEET (*RRRRRRRRRRRGGGMAGPPHIVEETGPHVI*) peptides were synthesized and HPLC-purified (>95%) at the School of Chemistry and Chemical Engineering, Shandong University. VIVIT and VEET were dissolved in DMSO and stored as stock solutions (20 mM) at −80°C. The final concentration of CsA and VIVIT/VEET used to treat keratinocyte cultures *in vitro* was 5 μm.

### Virus Infection

For virus infection, keratinocytes were seeded at a density of 4 × 10^5^ cells/well in six-well plates and incubated for 24 h, after which the cells were infected either with an oncogenic H-Ras^*G12V*^ expressing retrovirus, a NFATc1 expressing lentivirus or a corresponding control virus for 6 h. After infection, the cells were cultured in growth medium for the desired times as indicated in each Figure Legend before being collected for analysis. Detailed procedures for virus preparation and infection followed the protocols described previously (Wu et al., 2010, 2018).

### Small Interfering RNA Transfection

Small interfering RNA (siRNA) transfections were performed as described previously (Chang et al., 2018). Briefly, culture-expanded human keratinocytes were transfected with a siRNA calcineurin B1, a siRNA NFATc1 or a scrambled siRNA (control) at a final concentration of 20 nM using Lipofectamine 3000 according to the manufacturer’s instructions. At 72 h, the cells were collected for RT-PCR analysis and for Western blot analysis. The oligo sequences of siRNAs used are listed in Supplementary Table 1.

### Visualization and Analysis of Intracellular Autophagic Vacuoles

Two approaches were used to detect autophagic vesicles: (1) LysoTracker Green DND-26 (Cell Signaling, Danvers, MA, United States, #8783) staining followed the protocol provided by the manufacturer. The dye was diluted (1:20,000) directly into normal growth media for a working concentration of 50 nM and analyzed immediately using a fluorescence microscope; (2) monodansylcadaverine (MDC) staining (Cayman, Ann Arbor, MI, United States, #600140), a fluorescent dye known as a specific marker for autophagic organelles, was used to treat cells at 50 mM for 30 min (Lin et al., 2014). After incubation, the cells were washed three times with PBS and were immediately analyzed by fluorescence microscopy.

### Electron Microscopy

The electron microscopy (EM) assay protocol was as previously described (Altman et al., 2014). Briefly, human keratinocytes infected with the H-Ras^*G12V*^ expressing retrovirus were collected at 24 h after infection and were treated in BD fixation/permeabilization Solution (BD Bioscience, Franklin Lake, New Jersey, United States, #554722) at 4°C for 30 min. They were then fixed in 0.1 M sodium cacodylate buffer pH 7.3 containing 2% paraformaldehyde and 2.5% glutaraldehyde and embedded in resin mixture of embed 812 and araldite. Seventy nm thin sections were cut from the fixed samples and poststained with 3% uranyl acetate and Reynolds lead citrate, then examined using a FEI Tecnai Spirit G2 transmission electron microscope (TEM); digital images were captured using a FEI Eagle camera.

### *In vivo* Assay (Xenograft)

Xenografts were performed as previously described (Wu et al., 2010; Zheng et al., 2010). Briefly, human keratinocytes were infected with a H-Ras^*G12V*^ expressing retrovirus or a control retrovirus. Six hours after infection, cells were collected and injected (10^6^ cells per injection, two side flank injections per mouse) into the dermal-epidermal junction of 8-week-old female NU/NU mice (Charles River, Wilmington, MA, United States). At 24 h after the injection, mice were given I.P. injections, every other day of 10 mg/kg DMSO (negative control), VEET (control peptide for VIVIT), or VIVIT. One week later, the grafts were collected for RT-PCR and western-blot analysis of autophagy-associated genes. All animal procedures were performed in accordance with National and International Animal Welfare Regulations and with approval from the Stomatology School of Shandong University Committee on Animal Care (No. GR201720, Date: 02-27-2017).

### Quantitative Reverse Transcription Polymerase Chain Reaction (RT-PCR) Assay

Total RNAs of primary human keratinocytes were extracted using the RNAeasy kit (Qiagen, Germantown, MD, United States, #74104) and were reverse transcribed to complementary DNA (cDNA) using a superscript III first strand kit (Bio-Rad, Hercules, CA, United States, 1708891). PCR reactions were performed with Takara SYBR^*R*^ Premix Ex Taq^TM^ II (Takara Bio Inc., Mountain View, CA, United States, #RR820B) using a LightCycler^*R*^ 480 II (Roche Diagnostics, Risch-Rotkreuz, Switzerland). One hundred ng of each cDNA in a total of 20 μl qRT-PCR reaction were used for amplification. PCR reactions followed the following procedures: 95°C for 30 s, 40 cycles at 95°C for 5 s, at 60°C for 20 s and ended with an elongation step for 15 s at 72°C. Ct values were used for quantification and relative mRNA expression levels were calculated using the 2^–ΔΔ*Ct*^ method normalized by the human housekeeping gene 36β4. Oligo sequences of each gene for PCR analysis are listed in Supplementary Table 2.

### Western Blot Analysis

Total proteins of primary human keratinocytes were extracted using the RIPA reagent [RIPA lysis buffer containing 1% PMSF (Solarbio, Beijing, China, #P0100) and 1% phosphatase inhibitor]. Determination of protein concentrations was made using a BCA protein assay kit (Solarbio, Beijing, China, #PC0020). The extracted proteins were separated by 12% SDS-PAGE gel electrophoresis and transferred to PVDF membranes (Merck Millipore Ltd., Darmstadt, Germany, #IPVH00010). The membranes were blocked with 5% milk dissolved in TBST (TBS powder and 0.1% Tween 20; pH 7.5) for at least 1 h, then incubated overnight with the primary antibody at 4°C. The primary antibodies, rabbit monoclonal anti-NFATc1 antibody (Cell Signaling, Danvers, MA, United States, #8032), rabbit monoclonal anti-NFATc4 antibody (Abcam, Cambridge, MA, United States, ab3447),anti-Gapdh antibody (Cell Signaling, Danvers, MA, United States, #5174), rabbit monoclonal anti-Beclin-1 antibody (Cell Signaling, Danvers, MA, United States, #3495), rabbit monoclonal anti-p62 antibody (Abcam, Cambridge, MA, United States, #ab109012),rabbit monoclonal anti-H-RAS antibody (Abcam, Cambridge, MA, United States, #ab32417), rabbit monoclonal anti-Laminin B antibody (Abcam, Cambridge, MA, United States, #ab109293), rabbit monoclonal anti-Lamp-1 antibody (Cell Signaling, Danvers, MA, United States #9091), rabbit monoclonal anti-CnB1 antibody (Abcam, Cambridge, MA, United States, #ab231630), rabbit monoclonal antiATG5-12 (complex) antibody (Bio-Rad, Hercules, CA, United States, #AAM79), rabbit monoclonal anti-LC3A/B antibody (Thermo Fisher Scientific, Waltham, MA, United States, #PA1-16931). The membranes were washed three times with TBST buffer for 5 min and incubated with Either goat anti-mouse or goat anti-rabbit IgG HRP antibodies(Cell Signaling, Danvers, MA, United States, #7074 and #7076) for 1 h at room temperature. Protein bands were observed using an ECL chemiluminescence system (Solarbio, Beijing, China, #SW2010) and analyzed using ImageJ software.

### Chromatin Immunoprecipitation Assay

Chromatin immunoprecipitation (ChIP) assays were carried out following the EpiQuik^TM^ ChIP Kit protocol (Epigentek, Farmingdale, NY, United States, #P-2002). Briefly, 1 × 10^6^ human keratinocytes were cross-linked with 1% formaldehyde, sonicated, precleared and then incubated with 2–5 mg antibody per reaction [rabbit monoclonal anti-NFATc1 antibody (1:100), Cell Signaling, Danvers, MA, United States, #8032]. The pulled-down complexes were washed with low and high salt buffers, and the DNA was extracted and precipitated. The enrichment of the DNA template was analyzed by qRT-PCR, using primers specific for the promoter region of the H-Ras gene. The corresponding primers used are listed in Supplementary Table 3.

### Immunofluorescence Staining

After washing the samples with PBS, the cells were fixed in the culture plates with freshly prepared 4% paraformaldehyde solution for 15 min, followed by three washes with PBS, each for 5 min. Cells were permeabilized with 2 ml 0.2–0.5% Triton X-100 in PBS for 10 min, then washed three times with PBS for 5 min each. 2% BSA or 10% secondary antibody homologous serum was incubated for 30 min and then washed twice with PBS. The primary antibody (anti-rabbit NFATc1, Abcam, Cambridge, MA, United States, #ab25916) was left overnight at 4°C in a humidity chamber under dark conditions. Added secondary antibody [DyLight594 goat anti-rabbit IgG(H+L), Multi Science, Hangzhou, China] and incubated at room temperature for 30–45 min, followed by four washes with PBS for 5 min each. Each slide was incubated with 0.5 ug/ml 4′,6-diamidino-2-phenylindole (DAPI, Abcam, Cambridge, MA, United States, #ab228549) for 10 min, then washed three times with PBS to remove excess DAPI. Twenty 20 μl blocker was added to seal the specimens and images were observed in a fluorescence microscope.

### Statistical Analysis

GraphPad Prism 7 (Graph Pad Software Inc., La Jolla, CA, United States) was used for statistical analyses and data are presented as means ± standard deviation (SD). Each experiment was performed at least three times. When comparing two groups, Student’s *t*-test was used, otherwise one-way or two-way ANOVA analysis was used. A *p*-value less than 0.05 is considered to be statistically significant, which is indicated with “^∗^” in the figures.

## Results

### Overexpression of H-Ras^*G12V*^ Induces Autophagy in Keratinocytes

**FIGURE 1:**
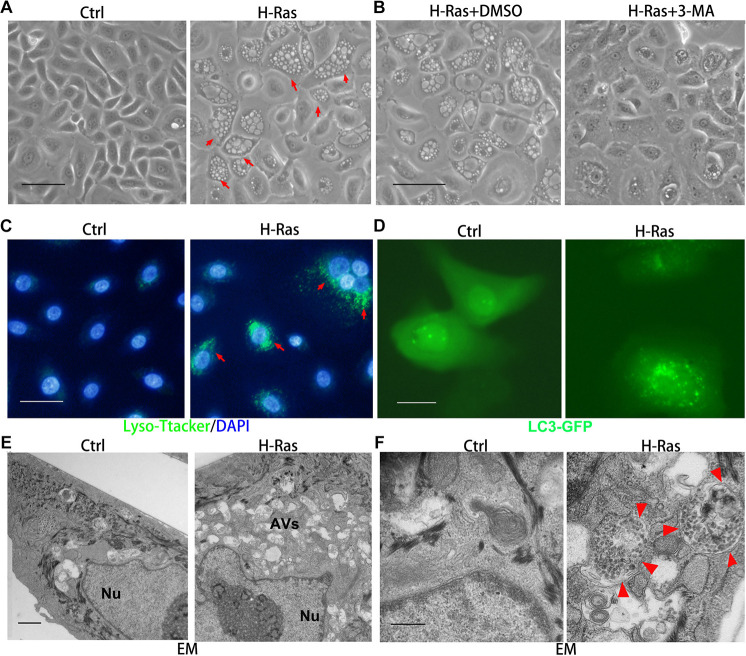
H-Ras induces autophagic vacuoles in human keratinocytes. **(A)** Human keratinocytes were infected with a H-Ras^*G12V*^ expressing or a control retrovirus. Representative cell images 24 h after infection are shown, red arrows indicate cells with bubbles. **(B)** Representative images of human keratinocytes at 24 h after infection with a H-Ras^*G12V*^ expressing retrovirus plus treatment with DMSO as a control or with a PI3K inhibitor (3-MA). **(C)** Human keratinocytes were infected with a H-Ras^*G12V*^ expressing or a control retrovirus, at 24 h after infection, LysoTracker Green was added into the growth medium plus DAPI (blue for nuclei) for 5 min, then examined using a fluorescence microscope. **(D)** Human keratinocytes were transfected with a LC3-GFP expression vector, and after transfection, the cells were infected with a H-Ras^*G12V*^ expressing or a control retrovirus. At 24 h after infection, the cells were checked for GFP expression using a fluorescence microscope. **(E,F)** Human keratinocytes were infected with a H-Ras^*G12V*^ expressing or a control retrovirus. At 24 h after infection, the cells were processed for electron microscopic (EM) analysis. Low magnification images are shown in panel **(E)**, and high magnification images are shown in panel **(F)**. Nu: nuclei, AVs: autophagic vesicles, red arrows indicate the double membrane structure of autophagosomes. **(A–D)** Bars = 50 mm, **(E)** bar = 2 mm, and **(F)** bar = 100 nm.

To test whether the oncogene H-Ras can induce autophagy in keratinocytes, we infected human primary keratinocytes with a H-Ras^*G12V*^ expressing and with a control retrovirus, and 24 h later, we observed the formation of many bubbles (red arrows) in ∼70% of keratinocytes infected with the H-Ras^*G12V*^ expressing virus, and the morphology of keratinocytes with bubbles was similar to cells containing autophagic vacuoles (Figure 1A). In order to confirm that the bubbles in H-Ras^*G12V*^ expressing cells were autophagic vacuoles, four approaches were carried out as follows: First, we used a PI3 kinase inhibitor, 3-MA, which is commonly used as an autophagy inhibitor, to treat keratinocytes immediately after infection with the H-Ras^*G12V*^ expressing virus. We observed that the bubbles induced in keratinocytes infected with the H-Ras^*G12V*^ expressing virus were significantly reduced by the treatment with 3-MA (Figure 1B), which suggests that these bubbles are autophagic vacuoles. Secondly, the maturation and degradation of autophagosomes involves fusion with endosome-lysosomes to form autolysosomes and the degradation of the inner membrane together with its luminal contents. Therefore, the activation of lysosomes can be observed during the formation of autophagosomes, which can be monitored using LysoTracker Green, a cell-permeable dye that fluoresces in acidic organelles. We incubated keratinocytes infected with the H-Ras^*G12V*^ expressing virus with LysoTracker Green, and found that the number of LysoTracker Green labeled keratinocytes was significantly increased in the keratinocytes infected with the H-Ras^*G12V*^ expressing virus (Figure 1C, red arrows). The activation of lysosomes in Ras expressing keratinocytes was further confirmed by staining with MDC, another marker of acidic components (Supplementary Figure 1). Thirdly, LC3, a protein associated with the membranes of autophagosomes, has been used to label autophagosomes. We transfected keratinocytes with a LC3-GFP expression vector followed by infection with the H-Ras^*G12V*^ expressing virus. At 24 h after infection, green punctate structures appeared in the H-Ras^*G12V*^ expressing keratinocytes, but not in the control cells (Figure 1D). These data suggested the formation of autophagosomes in H-Ras^*G12V*^ expressing keratinocytes. Finally, we investigated the fine structure of Ras-expressing keratinocytes using TEM. That ultrastructural analysis revealed that keratinocytes without H-Ras expression contain a limited number of vesicles with normal structures and the size of those cells was also smaller (Figures 1E,F). In contrast, massive vacuoles (AVs) appeared in the H-Ras^*G12V*^ expressing keratinocytes. The typical structure of autophagosomes, which contains both bilaminar membranes and microvesicular membrane fragments coupled with an intercellular localization were frequently observed in H-Ras^*G12V*^ expressing cells (Figure 1F, red arrows). They also contained electron-dense granular materials coupled with lysosome contents (Figure 1F, red arrows). Taken together, these data show that the stable expression of H-Ras^*G12V*^ can significantly induce autophagy in human keratinocytes.

### Inhibition of Calcineurin/NFAT Signaling Inhibits H-Ras^*G12V*^ Induced Autophagy *in vitro*

**FIGURE 2:**
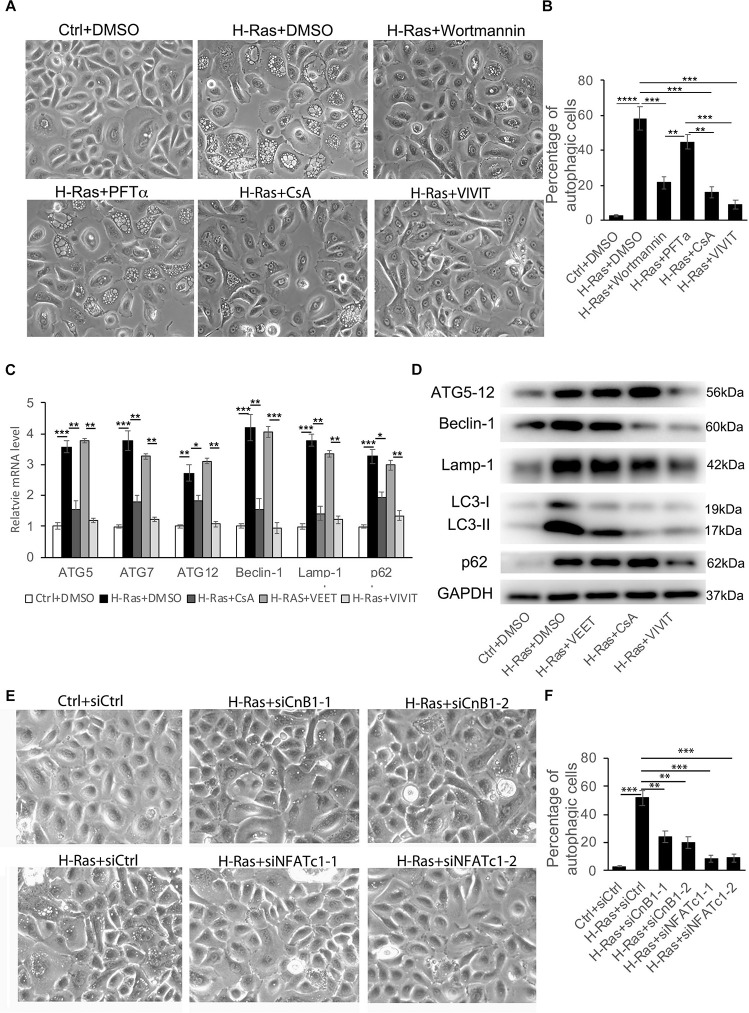
H-Ras induced autophagy is suppressed by inhibition of the calcineurin/NFAT pathway. **(A)** Human keratinocytes infected with a H-Ras^*G12V*^ expressing or a control retrovirus were treated with different inhibitors as indicated, DMSO as a negative control. Representative cell images 24 h after treatment are shown. **(B)** Quantification analysis of the percentage of cells with bubbles from panel **(A).** **(C)** Human keratinocytes infected with a H-Ras^*G12V*^ expressing or a control retrovirus were treated with the calcineurin/NFAT inhibitors CsA or VIVIT, or with VEET as a control peptide for VIVIT for 24 h, then cells were collected for quantitative RT-PCR analysis of autophagy-associated genes as indicated. **(D)** Human keratinocytes were treated with the same conditions as shown in panel **(C)**, then were lysed for immunoblotting analysis of autophagy-associated genes as indicated, GAPDH as a loading control. **(E)** Human keratinocytes were transfected with two independent siRNAs of CnB1 (siCnB1-1 and siCnB1-2) or NFATc1 (siNFATc1-1 and siNFATc1-2) or scramble siRNAs (siCtrl) as a control, and 2 days later, the cells were infected with a H-Ras^*G12V*^ expressing or a control retrovirus. Representative cell images 24 h after infection are shown. **(F)** Quantification analysis of the percentage of cells with bubbles from panel **(E)**. **(B,C,F)** All experiments were carried out three times, and error bars represent means ± SD; *p* values are indicated with “*,” *p < 0.05, **p < 0.01, ***p < 0.005 when comparing two corresponding groups indicated with the black lines by Student’s *t*-test.

Our previous study showed that the overexpression of H-Ras^*G12V*^ can induce the differentiation of human keratinocytes and both the p53 and the calcineurin/NFAT pathways are involved (Wu et al., 2010). Therefore, we hypothesized that the p53 and/or calcineurin/NFAT pathways are potentially involved in the regulation of H-Ras induced autophagy. To test that hypothesis, we infected human keratinocytes with a H-Ras^*G12V*^ expressing virus, then treated them with different inhibitors, 10 μM PFTα (a p53 inhibitor) (Park and Koh, 2019), 5 μM Cyclosporin A (CsA, a calcineurin inhibitor) (Zhou et al., 2021), 2 μM VIVIT (a NFAT inhibitor) (Wu et al., 2010), 100 nM wortmannin (a PI3K inhibitor) (Meyer et al., 2018) as a positive control and DMSO as a negative control. We found that treatment with the positive control, wortmannin (H-Ras+wortmannin) significantly inhibited the formation of autophagic vacuoles in H-Ras overexpressing keratinocytes (Figure 2A), compared with the negative control treated with DMSO (H-Ras+DMSO). Surprisingly, treatment with the p53 inhibitor (H-Ras+PFTα) didn’t significantly block the vacuole formation, but treatment with CsA (H-Ras+CsA) or with VIVIT (H-Ras+VIVIT), which inhibits the calcineurin/NFAT pathway, profoundly suppressed vacuole formation in H-Ras overexpressing keratinocytes (Figure 2A). Quantification of that analysis showed that calcineurin/NFAT inhibitors efficiently blocked the formation of autophagic vacuoles, and the efficiency of blocking vacuole formation was highest in VIVIT treated cells among all the groups (Figure 2B). To further confirm that the overexpression of H-Ras^*G12V*^ can induce autophagic formation, and that induction can be blocked by inhibition of the calcineurin/NFAT pathway, we used RT-PCR to check the mRNA expression levels of autophagy-associated genes in human keratinocytes with H-Ras^*G12V*^ overexpression treated with or without CsA, VIVIT or VEET (a negative control peptide of VIVIT). We found that the overexpression of H-Ras^*G12V*^ significantly induced the mRNA expression levels of autophagy-associated genes including ATG5, ATG7, ATG12, Beclin-1, Lamp-1, and p62 (Figure 2C), which further supported the observation of Ras-inducing autophagy formation shown in Figure 1. The increased expression of autophagy-associated genes was abolished by treatment with CsA or VIVIT, but not with the negative control peptide VEET (Figure 2C). This result was further validated by immunoblotting analysis of the levels of autophagy-associated proteins, especially significantly down-regulated by VIVIT treatment (Figure 2D). To further verify that inhibition of the calcineurin/NFAT pathway suppresses H-Ras^*G12V*^ induced autophagic activity, we used siRNA to knockdown either CnB1 (siCnB1) or NFATc1 (siNFATc1), and the knockdown efficiencies were confirmed by real time PCR and immunoblotting (Supplementary Figure 2). Similar to the effect of treatment with CsA or VIVIT, we observed that the siRNA knockdown of CnB1 or NFATc1 significantly reduced the number of autophagic vacuoles induced by H-Ras^*G12V*^ overexpression (Figures 2E,F). These data show that the inhibition of calcineurin/NFAT signaling blocked the autophagy induced by overexpression of H-Ras^*G12V*^ in human keratinocytes *in vitro*.

### Inhibition of Calcineurin/NFAT Signaling Suppresses H-Ras Induced Autophagy *in vivo*

**FIGURE 3:**
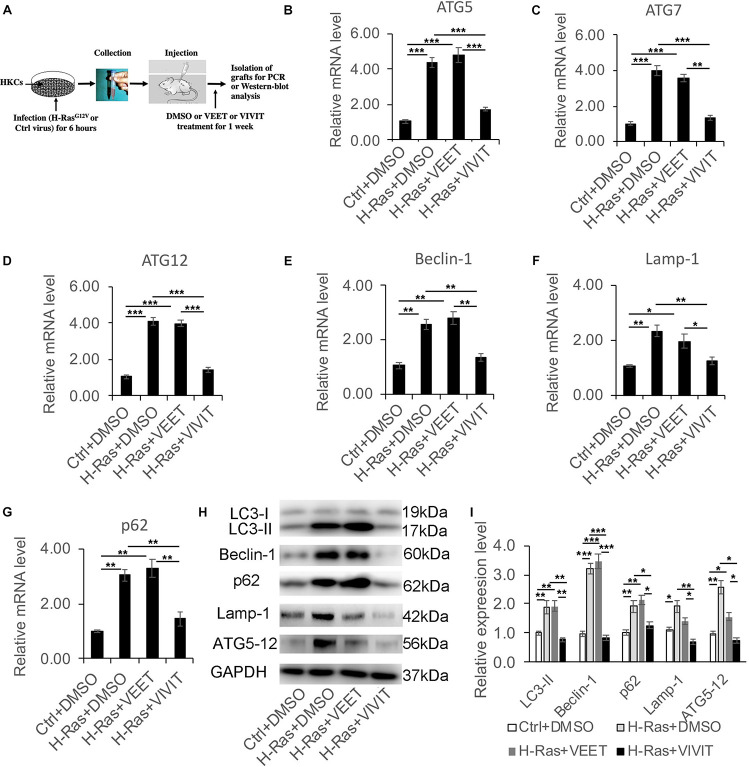
Nuclear factor of activated T cells inhibitor VIVIT suppresses the increased expression of autophagy-associated genes induced by the overexpression of H-Ras *in vivo*. **(A)** Scheme of the *in vivo* assay: Human keratinocytes (HKCs) were infected with a H-Ras^*G12V*^ expressing or a control retrovirus for 6 h, then were subcutaneously injected into the dorsal skin of NU/NU mice, after which the mice were treated with DMSO, VEET, or VIVIT by intraperitoneal injection. One week later, the grafts were collected for RT-PCR and western-blot analysis of autophagy-associated genes. **(B–G)** RT-PCR analysis of autophagy-associated genes as indicated from the grafts collected as shown in panel **(A).** **(H)** The grafts collected were lysed for immunoblotting analysis of autophagy-associated genes LC3, Beclin-1, p62, Lamp-1, and ATG5-12, GAPDH as a loading control. **(I)** Quantification of western-blot analysis shown in panel **(H)** for the relative expression levels of LC3, Beclin-1, p62, Lamp-1, and ATG5-12. Four mice (*n* = 4) were used in each group, and error bars represent means ± SD; *p* values are indicated with “*,” *p < 0.05, **p < 0.01, ***p < 0.005 when comparing two corresponding groups indicated with the black lines by Student’s *t*-test.

Next, we assessed whether the induction of autophagy by H-Ras^*G12V*^ could be blocked by the inhibition of calcineurin/NFAT signaling *in vivo*. We infected human keratinocytes with a H-Ras^*G12V*^ expressing and with a control retrovirus, then the infected human keratinocytes were subcutaneously injected into the dorsal skin of nu/nu mice. Those mice were then treated with DMSO, VIVIT or VEET once every 2 days, and 1 week after the injection, the grafts were collected for RT-PCR and western-blot analysis to characterize the expression of autophagy-associated genes (Figure 3A). The RT-PCR analysis demonstrated the significantly increased expression of all autophagy-associated genes tested, including ATG5, ATG7, ATG12, Beclin-1, Lamp-1, and p62, caused by the overexpression of H-Ras^*G12V*^. The induction of those autophagic genes was suppressed by treatment with VIVIT, but not by treatment with the control peptide VEET (Figures 3B–G). The western blot analysis of LC3, Beclin-1, p62, Lamp-1, and ATG5-12 proteins further supported the results of the RT-PCR analysis (Figures 3H,I). These results suggested that the inhibition of calcineurin/NFAT signaling by VIVIT could block autophagy formation caused by the overexpression of H-Ras^*G12V*^, supporting the results of the *in vitro* study.

### Overexpression of H-Ras^*G12V*^ Activates the Calcineurin/NFAT Pathway in Human Keratinocytes

**FIGURE 4:**
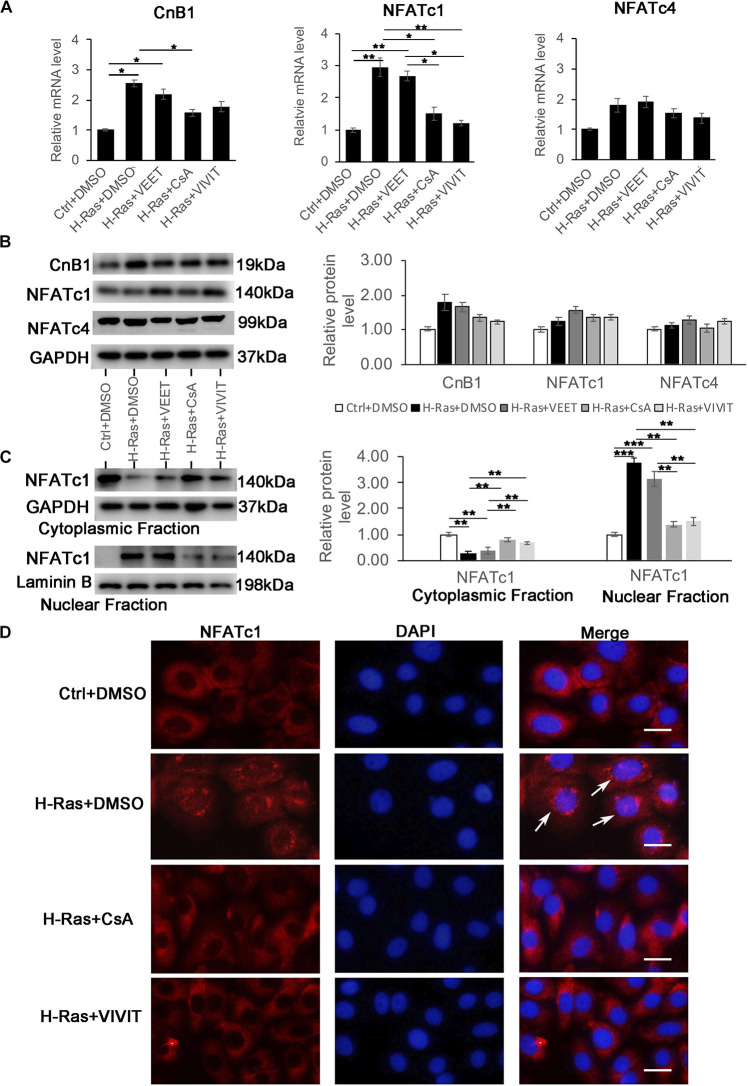
Overexpression of H-Ras induces the nuclear translocation of NFATc1A. Human keratinocytes infected with a H-Ras^*G12V*^ expressing or a control retrovirus were treated with the calcineurin/NFAT inhibitors CsA, VIVIT, VEET as a control peptide for VIVIT or DSMO as negative control for 24 h, after which the cells were collected for quantitative RT-PCR analysis of calcineurin B1 (CnB1), NFATc1, and NFATc4 expression. **(B)** (Left panel) human keratinocytes were treated with same conditions as shown in **(A)**, then cells were lysed for immunoblotting analysis of calcineurin B1 (CnB1), NFATc1, and NFATc4 expression, GAPDH as a loading control. (Right panel) Quantification of the relative levels of CnB1, NFATc1 and NFATc4 in the left panel. **(C)** (Left panel) human keratinocytes treated with same conditions as shown in panel **(A)** 24 h after infection, the cells were lysed for preparation of cytoplasmic and nuclear fractions. Both fractions were immunoblotted with the NFATc1 antibody, GAPDH as a loading control for the cytoplasmic fraction, and Laminin B as a loading control for the nuclear fraction. Right panel: Quantification of the relative levels of NFATc1 in the left panel. **(A–C)** All experiments were carried out three times, and error bars represent means + SD; *p* values are indicated with “*,” *p < 0.05, **p < 0.01, ***p < 0.005 when comparing two corresponding groups indicated with the black lines by Student’s *t*-test. **(D)** Human keratinocytes infected with a H-Ras^*G12V*^ expressing or a control retrovirus treated with the calcineurin/NFAT inhibitors CsA and VIVIT or DMSO as a control for 24 h, followed by immunofluorescence staining with the NAFTc1 antibody (red), and DAPI stain for nuclei. Merged images (Merge) are shown in the right column, and white arrows indicate the nuclear staining of NFATc1. Bars = 50 μm.

The inhibition of calcineurin/NFAT signaling by CsA or VIVIT could block the formation of autophagy induced by H-Ras^*G12V*^, indicating that this pathway was potentially activated by the H-Ras involved autophagic formation in human keratinocytes. To test that hypothesis, we first checked if the overexpression of H-Ras^*G12V*^ could directly induce the expression of calcineurin B1 (CnB1), the only calcineurin B isoform in keratinocytes (Mammucari et al., 2005) and/or the NFAT isoforms C1 (NFATc1) and C4 (NFATc4), which have been mainly studied in human keratinocytes (Zhou et al., 2021). Analysis of mRNA and protein expression levels revealed that the overexpression of H-Ras^*G12V*^ in keratinocytes led to a certain upregulation of CnB1 and NFATc1 at mRNA level, but not dramatical change for protein level of CnB1 and NFATc1, and there was no significant change of both mRNA and protein level of NFATc4 (Figures 4A,B). Next, we investigated whether the overexpression of H-Ras^*G12V*^ could activate calcineurin/NFATc signaling. It has been shown that activation of the calcineurin/NFATc signaling pathway leads to the nuclear translocation of NFATc, which is usually used as a marker to assess whether the calcineurin/NFAT signaling is activated. Therefore, we tested whether H-Ras could affect the nuclear translocation of NFATc1 by immune-blotting analysis of the nuclear fraction derived from H-Ras^*G12V*^ overexpressing keratinocytes. The immune-blotting analysis showed that the overexpression of H-Ras significantly increased the NFATc1 level in the nuclear fraction, but decreased it in the cytoplasmic fraction (Figure 4C), suggesting that H-Ras overexpression enhanced the nuclear translocation of NFATc1. Importantly, the increased level of NFATc1 in the nuclear fraction elicited by H-Ras was significantly abolished by inhibition of the calcineurin/NFAT signal pathway with CsA or VIVIT, but not by treatment with the negative control VEET (Figure 4C). To further confirm this finding, we performed immunofluorescence staining of NFATc1 in keratinocytes infected with H-Ras^*G12V*^ expressing virus together with treatment of CsA and VIVIT. We found that the overexpression of H-Ras^*G12V*^ could enhance the nuclear translocation of NFATc1 (Figure 4D, white arrows), which was blocked by treatment with CsA or VIVIT. Taken together, these data suggested that H-Ras could enhance the nuclear translocation of NFATc1, indicating the activation of the calcineurin/NFAT signaling pathway by H-Ras in human keratinocytes.

### The Activation of Calcineurin/NFAT Signaling Doesn’t Induce Autophagy in Keratinocytes

**FIGURE 5:**
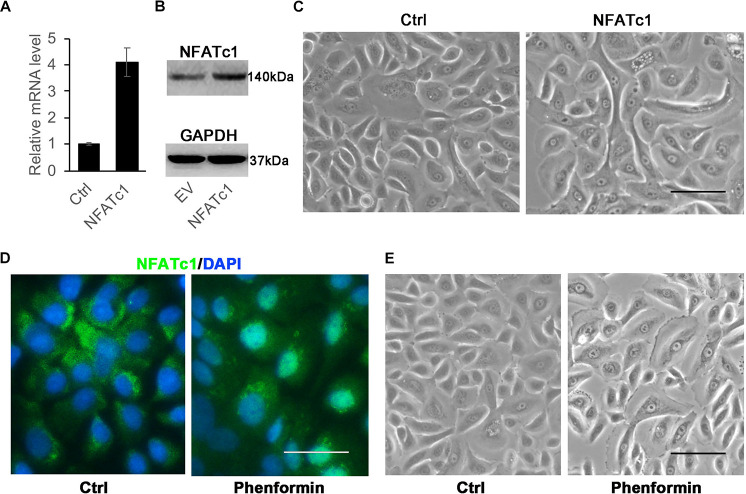
Activation of NFATc1 doesn’t significantly induce vacuole formation in human keratinocytes**. (A,B)** Human keratinocytes were infected with a NFATc1 expressing or a control retrovirus, and 24 h after infection, the cells were collected for analysis of NFATc1 expression by RT-PCR **(A)** or by immunoblotting **(B)**. GAPDH as a loading control in panel **(B).** The experiments were carried out three times, and error bars represent means ± SD; *p* values are indicated with “*,” **p < 0.01, when comparing with the control group by Student’s *t*-test in panel **(A)**. **(C)** Representative images of human keratinocytes infected with a NFATc1 expressing or a control retrovirus for 24 h. **(D,E)** Human keratinocytes were treated with 1 mM phenformin for 24 h, then were fixed and analyzed NFATc1 (green) expression by immunofluorescence staining, DAPI stained for nuclei (blue). Representative images of human keratinocytes are shown in panel **(E).** **(C–E)** Bars = 50 mm.

Since the overexpression of H-Ras^*G12V*^ could induce autophagy and also could activate calcineurin/NFAT signaling, we next asked if the activation of calcineurin/NFAT can induce autophagy in keratinocytes. To activate calcineurin/NFAT signaling, we used two approaches. First, we infected human keratinocytes with a NFATc1 expressing virus, and 24 h later, collected the cells and validated the stable expression of NFATc1 at both the mRNA and protein levels (Figures 5A,B), but we didn’t observe vacuole formation in those NFATc1 overexpressing cells (Figure 5C). We recently reported that phenformin, an AMPK activator, activates calcineurin/NFAT signaling to induce differentiation in keratinocytes (Zhou et al., 2021), and therefore, we treated keratinocytes with phenformin to activate the calcineurin/NFAT pathway. Immunofluorescence staining showed that indeed, treatment with phenformin significantly induced the nuclear translocation of NFATc1 (Figure 5D), but the formation of vacuoles was not observed (Figure 5E) in phenformin-treated keratinocytes. This result suggested that the activation of calcineurin/NFAT signaling without the overexpression of H-Ras^*G12V*^ doesn’t induce autophagy in keratinocytes.

### Inhibition of Calcineurin/NFATc Signaling Suppresses the Expression of H-Ras in H-Ras^*G12V*^ Overexpressing Keratinocytes

**FIGURE 6:**
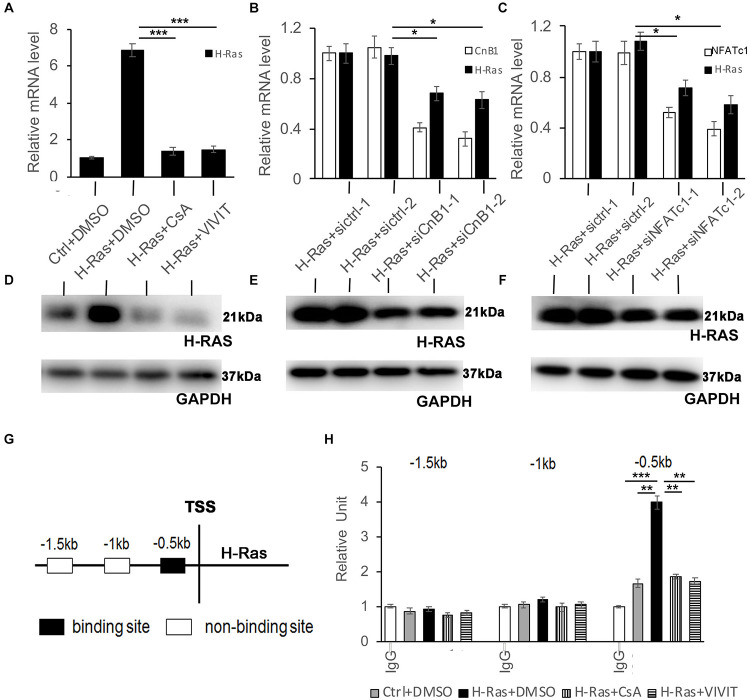
NFATc1 regulates H-Ras expression in human keratinocytes overexpressing H-RasA. Human keratinocytes were infected with a H-Ras^*G12V*^ expressing or a control retrovirus and were then treated with the calcineurin/NFAT inhibitors CsA, VIVIT, or DSMO as a negative control for 24 h, then were collected for quantitative RT-PCR analysis of H-Ras expression. **(B-C)** Human keratinocytes were transfected with two independent siRNAs of CnB1 (siCnB1-1 and siCnB1-2) in panel **(B)** or NFATc1 (siNFATc1-1 and siNFATc1-2) in panel **(C)** or scramble siRNAs (siCtrl) in both panels **(A,B)** as a control 2 days after transfection, the cells were infected with a H-Ras^*G12V*^ expressing or a control retrovirus, and 24 h later, the treated cells were collected for quantitative RT-PCR analysis of CnB1 **(B)** or NFATc1 **(C)**, and H-Ras expression. **(D–F)** Western-blot analysis of H-Ras protein levels in cells from panels **(A–C)**, respectively. GAPDH as loading control. **(G,H)** Three potential binding sites located upstream of the H-Ras transcriptional start site (TSS) as indicated in panel **(G)** were analyzed by CHIP assays, and CHIP products derived from human keratinocytes with different conditions as labeled were analyzed by RT-PCR in panel **(H)**. All experiments were carried out three times, and error bars represent means ± SD; *p* values are indicated with “*,” *p < 0.05, **p < 0.01, and ***p < 0.005 when comparing two corresponding groups indicated with the black lines by Student’s *t*-test.

Since the activation of calcineurin/NFAT signaling was not necessary to induce autophagy in keratinocytes, we wondered whether that pathway is upstream of H-Ras to affect autophagy formation induced by the overexpression of H-Ras^*G12V*^. To test that possibility, we checked whether the inhibition of calcineurin/NFAT signaling affected the expression of H-Ras in keratinocytes infected with the H-Ras^*G12V*^ expressing vector. We found that treatment with CsA or VIVIT significantly decreased the mRNA level of H-Ras in keratinocytes (Figure 6A). To validate this finding, we knocked down either CnB1 or NFATc1 using two independent siRNAs in H-Ras^*G12V*^ overexpressing keratinocytes (Figures 6B,C), and found that both siRNAs significantly suppressed the expression of H-Ras in keratinocytes (Figures 6B,C). Cells treated similarly as shown in Figures 6A–C were analyzed for the protein level of H-Ras, and western-blot analysis showed a result similar to the RT-PCR analysis (Figures 6D–F). These results suggested that calcineurin/NFAT signaling likely controls the expression of H-Ras in human keratinocytes.

To investigate whether H-Ras is a potential target of NFATc1, we first analyzed published NFATc1 CHIP-seq data^1^ and found that H-Ras is a putative target of NFATc1 in human endothelial cells and B lymphocytes (Gertz et al., 2013; Suehiro et al., 2014). Analysis of the 5 kb promoter region of H-Ras identified three potential binding sites predicted upstream of the translation start site of H-Ras (Figure 6G). To determine whether NFAT directly targets H-Ras in keratinocytes, CHIP assays were performed. RT-PCR analysis of the CHIP products showed that NFATc1 likely binds one of the three predicted binding sites, which is 0.5 kb upstream of the translation start site of the H-Ras gene (Figure 6G, black box), that was not detected by the control non-immune IgG (Figure 6H, IgG). Interestingly, the binding was weak under normal physiological conditions (Figure 6H, Ctrl+DMSO) but was clearly enhanced by the overexpression of H-Ras^*G12V*^ (Figure 6H, H-Ras+DMSO). Importantly, the enhanced binding caused by the overexpression of H-Ras^*G12V*^ was significantly abolished by the inhibition of calcineurin/NFAT signaling with CsA or VIVIT (Figure 6H, H-Ras+CsA or H-Ras+VIVIT). Together, these data suggest that during the overexpression of H-Ras, NFATc1 can bind the promoter region of H-Ras and affect its expression level in human keratinocytes.

## Discussion

The involvement of autophagy in cancer initiation and progression has drawn much attention over the past few years. Ras, a membrane-anchored protein, controls multiple signaling pathways involved in cell tumorigenicity (Elgendy et al., 2011; Guo et al., 2011; Schmukler et al., 2014). The crosstalk between Ras and autophagy has been studied extensively, especially in cancer cells. Ras has been shown to both positively and negatively regulate autophagy by controlling different downstream pathways, which depends on the cell type and cell context (Chi et al., 1999; Furuta et al., 2004; Berry and Baehrecke, 2007; Byun et al., 2009; Elgendy et al., 2011; Kim et al., 2011; Wu et al., 2011). Until now, the relationship between Ras and autophagy has not been elucidated in keratinocytes. Here, we demonstrate that the overexpression of H-Ras^*G12V*^ significantly induces autophagy in human primary keratinocytes, which is indirectly supported by a recently published paper that reported that RasGRP1, a Ras activator, induces autophagy in human keratinocytes (Fonseca et al., 2021). It has been shown that the regulation of autophagy by H-Ras is mainly through interactions with Rac1/MKK7/JNK or Raf-1/MEK1/2/ERK or the class III PI3K pathways (Schmukler et al., 2014). We found that a PI3K inhibitor could sufficiently block autophagy induced by the overexpression of H-Ras^*G12V*^ in human keratinocytes. However, the inhibition of p53 signaling, one of the key mediators controlling autophagy in tumor cells (Mrakovcic and Frohlich, 2018), was not able to suppress the H-Ras^*G12V*^ induced autophagy in keratinocytes, which suggests that p53 signaling may not be involved in autophagy formation induced by the overexpression of H-Ras^*G12V*^. Surprisingly, the inhibition of calcineurin/NFAT signaling either by the calcineurin inhibitor CsA or by the NFAT inhibitor VIVIT significantly suppressed keratinocyte autophagy induced by the overexpression of H-Ras^*G12V*^ both *in vitro* and *in vivo*. This result was further confirmed by the knockdown of calcineurin B1 or NFATc1 to block the calcineurin/NFAT pathway in H-Ras^*G12V*^ overexpressing human keratinocytes. This indicated that the calcineurin/NFAT pathway plays a crucial role in the regulation of autophagy in human primary keratinocytes by the oncogene H-Ras.

The calcineurin/NFAT signaling pathway has been shown to play an important role in controlling keratinocyte differentiation (Al-Daraji et al., 2002; Mammucari et al., 2005; Wu et al., 2010; Zhou et al., 2021). Our previous study reported that inhibition of the calcineurin/NFAT pathway either by CsA or VIVIT suppressed the oncogene H-Ras^*G12V*^ induced senescence and differentiation to promote keratinocyte tumorigenesis through inhibition of the p53 pathway (Wu et al., 2010). Calcineurin, which is activated by calcium signaling, has been shown to be involved in the regulation of autophagy mainly through its substrate transcriptional factor EB (TFEB), a master regulator of lysosomal biogenesis and autophagy (Urbanelli et al., 2014; Tong and Song, 2015), however, little is known about the role of the calcineurin classic substate NFAT (calcineurin/NFAT) associated with autophagy. A previous study has shown that active V12Ras can induce NFAT transcription activity in mast cells through the Rac1 pathway (Turner and Cantrell, 1997). Here, we found that the overexpression of H-Ras^*G12V*^ strongly enhanced the nuclear translocation of NFATc1 in human keratinocytes, suggesting that the oncogene mutant H-Ras can activate the calcineurin/NFAT signaling pathway. However, the underlying mechanism how H-Ras activates the calcineurin/NFAT signaling needs further investigation, since H-Ras^*G12V*^ induced both CnB1 and NFATc1 mRNA level, but not protein level. Interestingly, activation of the calcineurin/NFATc1 pathway, for instance by the overexpression of NFATc1 or by the nuclear translocation of NFATc1 induced by phenformin, was not able to induce autophagy in keratinocytes without the overexpression of H-Ras^*G12V*^. These data suggested that the calcineurin/NFAT pathway involved in autophagy in keratinocytes was under conditions of high expression of the oncogene mutation H-Ras.

Considering the highly efficient suppression of H-Ras^*G12V*^ induced autophagy by calcineurin/NFAT inhibitors, we suspected that the calcineurin/NFATc1 pathway possibly acts as an upstream factor to control H-Ras levels in human keratinocytes. Indeed, both CsA and VIVIT strongly reduced H-Ras levels in keratinocytes overexpressing H-Ras^*G12V*^, which suggested that calcineurin/NFATc1 signaling likely controls H-Ras expression. The crosstalk between calcineurin and Ras has been reported previously, for instance, carabin, an endogenous inhibitor of calcineurin, was shown to inhibit the Ras signaling pathway in T cells (Pan et al., 2007), and calcineurin and Ras have been shown to have synergistic effects in activating IL-2 and the transcription of other cytokine genes in T cells (Woodrow et al., 1993). Although whether calcineurin/NFAT signaling controls Ras expression has not been reported, analysis of a published CHIP-seq dataset indicated that H-Ras is a potential target of NFATc1 (see text footnote 1). Our CHIP assays demonstrated that NFATc1 could directly bind the promoter region of H-Ras, indicating that NFATc1 potentially controls H-Ras expression in human keratinocytes. Interestingly, the binding efficiency is low under normal physiological conditions, but is significantly enhanced by the overexpression of H-Ras, suggesting that NFATc1 could directly control H-Ras transcription in H-Ras^*G12V*^ overexpressing keratinocytes. Taken together, these data suggest that overexpression of the oncogene H-Ras can activate the calcineurin/NFATc1 pathway to induce the nuclear translocation of NFATc1 to bind the promoter of H-Ras and to control H-Ras expression in human keratinocytes. However, it is still difficult to explain why the expression level of H-Ras, especially at the protein level, was much lower in H-Ras^*G12V*^ overexpressing keratinocytes compared to cells with knockdown of calcineurin B1 or NFATc1 (Figure 6). Therefore, the underlying mechanism of how calcineurin/NFAT inhibitors control the H-Ras level in keratinocytes needs further investigation in the future.

The calcineurin/NFAT signaling pathway, originally identified in T cells to play an essential role in the regulation of immune responses, has been shown to play important biological functions involved in cell proliferation and differentiation in different types of cells (Crabtree and Olson, 2002; Hogan et al., 2003; Wang et al., 2015). More and more evidence has revealed that the calcineurin/NFAT signaling pathway plays a tumor suppressing function in cooperation with Ras pathways (Dotto, 2011). For example, NFAT1 (NFATc2) can cooperate with the Ras/Ras/MEK/ERK pathway to induce apoptosis in NIH3T3 fibroblasts, and the activation of NFAT1 can shift the oncogenic Ras pathway to act as a tumor suppressor (Robbs et al., 2013). Our previous study (Wu et al., 2010) demonstrated that inhibition of the calcineurin/NFAT pathway blocks keratinocyte senescence and differentiation induced by the overexpression of H-Ras. This study has shown that calcineurin/NFAT inhibitors suppress keratinocyte autophagy induced by the overexpression of H-Ras^*G12V*^. Our data suggest that the calcineurin/NFAT pathway can coordinate with the Ras pathway to play an important role as a fail-safe mechanism to maintain keratinocyte homeostasis under activation of the oncogene Ras pathway.

## Data Availability Statement

The raw data supporting the conclusions of this article will be made available by the authors, without undue reservation.

## Ethics Statement

The studies involving human participants were reviewed and approved by the use of human tissues collected from discarded hospital specimens without any personal identity information and the protocol (Protocol No. GR201711, Date: 02-27-2017), was approved by the Ethics Committee of the Hospital of Stomatology, Shandong University. Written informed consent for participation was not required for this study in accordance with the national legislation and the institutional requirements. The animal protocol used in this study were approved by the Ethics Committee of the Hospital of Stomatology, Shandong University (Protocol No. GR201720, Date: 02-27-2017).

## Author Contributions

SW, HQ, JG, JH, and XW conceived and designed the study, and provided acquisition, analysis and interpretation of data. SW, HQ, LZ, PL, DZ, QZ, FB, ZW, and YY collected the data and conducted the statistical analyses. JG, JH, and XW performed the development of methodology, writing, and review of the manuscript. All authors read and approved the final version of the manuscript.

## Conflict of Interest

The authors declare that the research was conducted in the absence of any commercial or financial relationships that could be construed as a potential conflict of interest.

## Footnotes

1 http://cistrome.org
