# Supplementary material for: Inhibition of Calcineurin/NFAT Signaling Blocks Oncogenic H-Ras Induced Autophagy in Primary Human Keratinocytes
Source: Front Cell Dev Biol. 2021 Jul 19;9:720111. doi: 10.3389/fcell.2021.720111 (PMC8328491; doi:10.3389/fcell.2021.720111)
Supplement: Supplementary file 1 [file Data_Sheet_1.PDF]

Supplementary Material

1 Supplementary Figures and Tables

1.1 Supplementary Figures

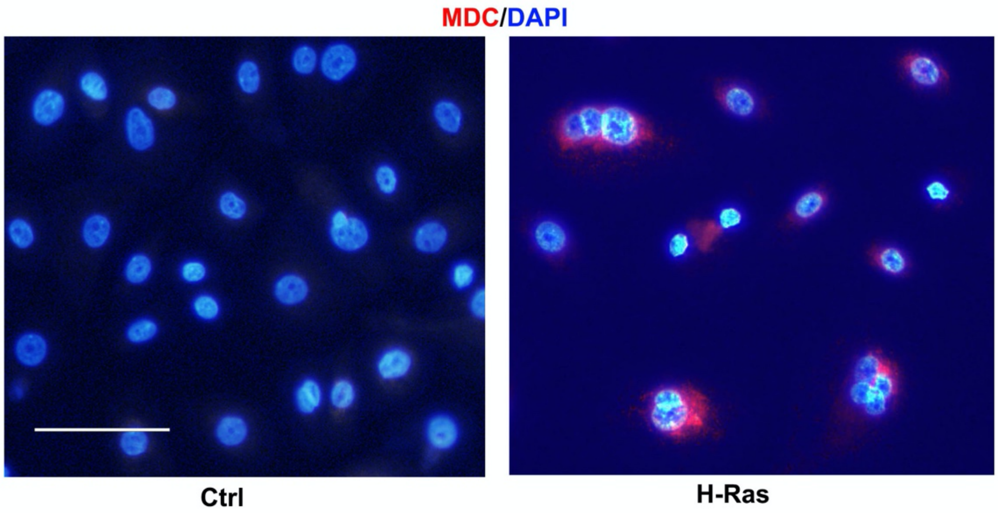

Supplementary Figure 1. Human keratinocytes were infected for 24 h with a H-Ras expressing or a control retrovirus. The dye Monodansylcadaverine (MDC, red) was then added into the growth medium plus DAPI to stain nuclei blue for 30 min, then washed three times with PBS and examined using a fluorescence microscope. Bars = 100 µm.

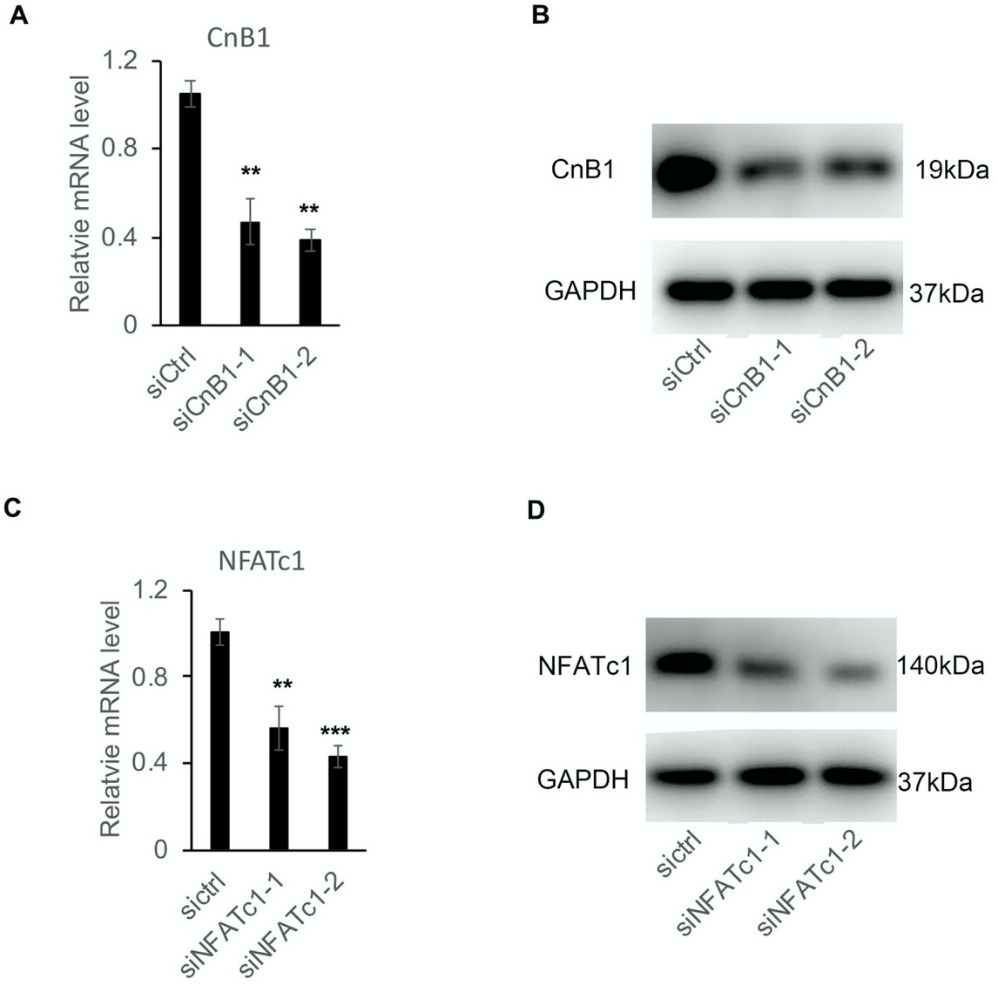

Supplementary Figure 2. Transfection of siRNAs efficiently inhibited the expression of CnB1 or NFATc1 in human keratinocytes

A-D: Human keratinocytes were transfected with two independent siRNAs of CnB1 (siCnB1-1 and siCnB1-2) or NFATc1 (siNFATc1-1 and siNFATc1-2) or scramble siRNAs (siCtrl) as a control, and 2 days later, the cells were collected for both qRT-PCR (**A,C**) and western-blot (**B,D**) analysis of CnB1 (**A,B**) or NFATc1 (**C,D**) expression. GAPDH as a loading control for western-blot analysis in **B** and **D**. All experiments were carried out 3 times, and error bars represent means $\pm$ SD; p values are indicated with "*", ** indicates P<0.01, *** indicates P<0.005 when comparing with control group (siCtrl) by Student's t test in **A** and **C**.

1.2 Supplementary Tables

Supplementary Table 1. Oligo sequences for siRNAs

Name	Sense Primer	Antisense primer
siCnB1-1	5'-GCA AGU UAU CCU UUG GAA ATT-3'	5'-UUU CCA AAG GAU AAC UUG CTT-3'
siCnB1-2	5'-CCU UUA GUA CAG CGA GUA ATT-3'	5'-UUA CUC GCU GUA CUA AAG GTT-3'.
siNFATc1-1	5'-GGGACCUGUGCAAGCCGAAUUCUCU-3'	AGAGAAUUCGGCUUGCACAGGUCCC-3'
siNFATc1-2	5'- CCCGUUCACGUCAGUUUCUACGUCU-3'	AGACGUAGAAACUGACGUGAACGGG-3'

Supplementary Table 2. PCR Primers used for qRT-PCR

Gene	Forward Primers	Reverse Primers
36β4	5'-GCAATGTTGCCAGTGTCTGT-3'	5'-GCCTTGACCTTTTCAGCAAG-3'
ATG5	5'- AGGGAAGCAGAACCAAGTAT-3'	5-CATTTCAGGTGTGCCTTCATA-3'
ATG7	5'-CAGTTTGCCCCTTTTAGTAGTGC-	5'-CCAGCCGATACTCGTTCAGC-3'
ATG12	5′-ATGACTAGCC-GGGACACC-3'	5′-CCAGTTTAC-CATCACTGCC-3′.
Beclin-1	5'-GCCCAGACAGGACTCTCTTAG-3'	5'-TGAACACACTTGCCAGTCTTC-3'
p62	5′-TGCCCAGACTACGACTTGTG-3′	5′-AGTGTCCGTGTTTCACCTTCC-3′
Lamp-1	5'-CAGATGTGTTAGTGGCACCCA-3'	5'-TTGGAAAGGTACGCCTGGATG-3'
Calcineurin B1	5'-TGAAGATGATGGTGGGGAAC-3'	5'-TTGTGGATATCTAGGCCACC-3'
NFATc1	5'-CAACGGTAACGCCATCTTTC-3'	5'-GACGTCGTTTCTGCGTCTTT-3'
NFATc4	5'-TTGCACTTGGGAAACTTGCC-3'	5'-TGCTCTCCTGAGTACTTG CT-3'
H-Ras	5'-CAGATCAAACGGGTGAAGGAC-3'	5'-GCCTGCCGAGATTCCACAG-3'

Supplementary Table 3. Primers used for qRT-PCR analysis of CHIP products

Name(region)	Forward Primers	Reverse Primers
NFATc1 (0.5kb)	5'-GAGGCGGAGCCAGTAGGG-3'	5'-CAGGGAGGGCCTCTGGTG-3'
NFATc2 (1.0kb)	5'- GCCTGGCCAACATGATGAAA-3'	5-TTGCCTGACTGTAAAACGCC-3'
NFATc1 (1.5kb)	5'-GTCGCAGTCAGAGCTCGG-3'	5'-TTCGGTTGGAAGTGAGGCC-3'
